# Supplementary material for: Dual-bionic superwetting gears with liquid directional steering for oil-water separation
Source: Nat Commun. 2023 Jul 12;14:4128. doi: 10.1038/s41467-023-39851-1 (PMC10338494; doi:10.1038/s41467-023-39851-1)
Supplement: Supplementary file 1 — Supplementary Information [file 41467_2023_39851_MOESM1_ESM.pdf]

Supplementary Information

Dual-Bionic Superwetting Gears with Liquid Directional Steering for Oil-Water Separation

Zhuoxing LIU[1,2], Zidong ZHAN[1,2], Tao SHEN[3], Ning LI[1], Chengqi ZHANG[1,3], Cunlong YU[1,2], Chuxin LI[4], Yifan SI[5*], Lei JIANG[1,2], and Zhichao DONG[1,2*]

Affiliations

1 CAS Key Laboratory of Bio-inspired Materials and Interfacial Science, Technical Institute of Physics and Chemistry, Chinese Academy of Sciences, Beijing 100190, China.

2 School of Future Technology, University of Chinese Academy of Sciences, Beijing 100049, China.

3 Key Laboratory of Bio-Inspired Smart Interfacial Science and Technology of Ministry of Education, School of Chemistry, Beihang University, Beijing 100191, China

4 Suzhou Institute for Advanced Research, University of Science and Technology of China, Suzhou, Jiangsu 215123, China

5 Department of Biomedical Engineering, City University of Hong Kong, Hong Kong SAR 999077, China

This file includes:

Supplementary Figures 1-9

Supplementary Table 1-2

Legend for Supplementary Movies

Supplementary References

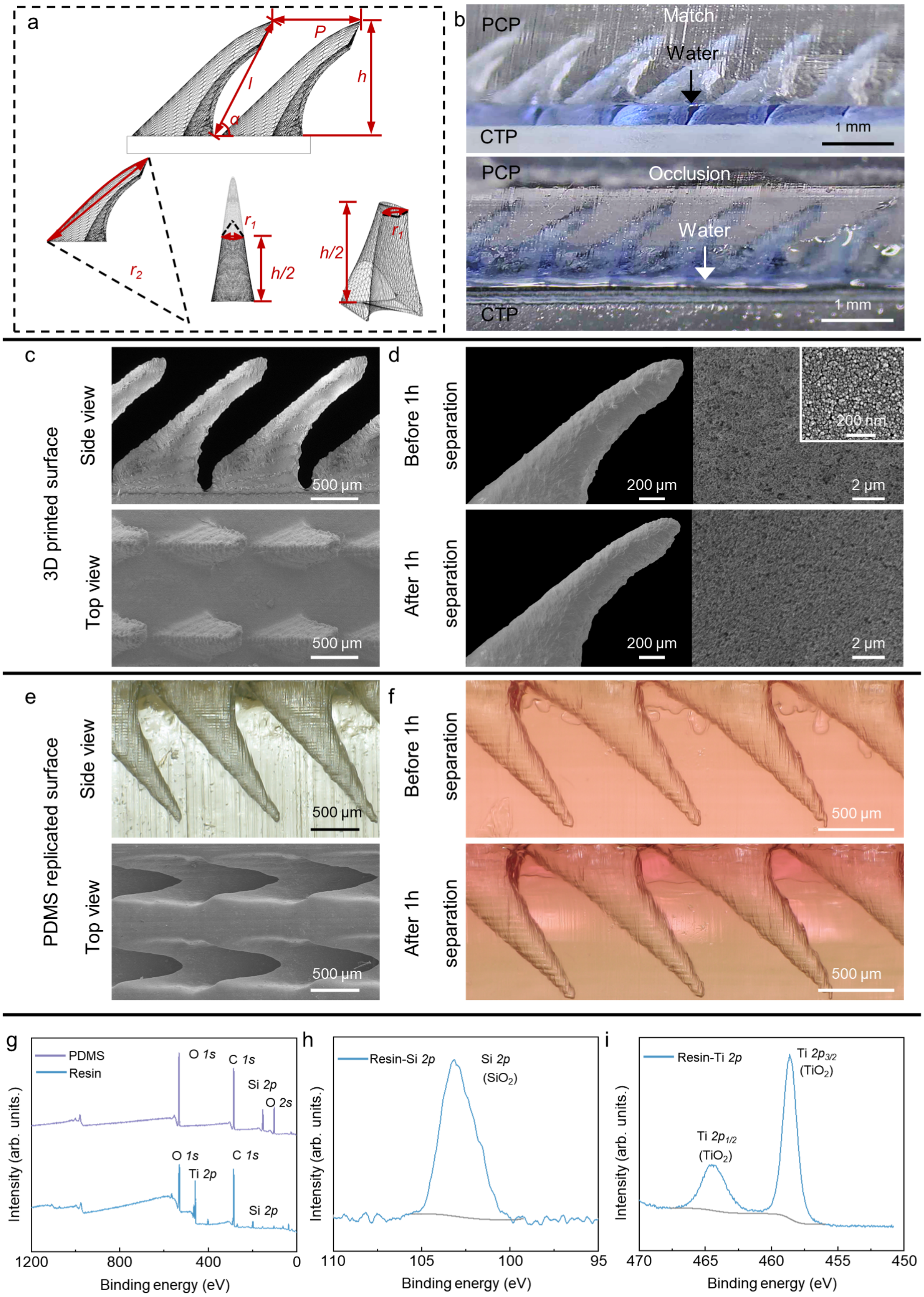

Supplementary Figure 1 | Surface morphologies of the 3D printed cat tongue plane (CTP) and replicating PDMS peristome-inspired cavity plane (PCP). a, Structural characteristics of the CTP model. The height (*h*), tip-to-tip pitch (*p*), length (*l*), and tilt angle (*a*) of ratchets. Each cat tongue-inspired tooth is endowed with the transverse curvature of radius r_1 and the longitudinal curvature of radius r_2. **b,** Occlusion between CTP and PCP enables water wicking both in and out of the surface plane. **c,** Microscope and SEM images of CTP from the side and

top views**. d,** Microscope image of CTG before separation and after separation. **e,** Microscope and SEM images of PCP from the side and top views**. f,** Microscope image of PCG before separation and after separation. **g,** XPS full spectrum of PDMS (purple) and resin (blue). **h,** Si *2p* pattern of resin surface. **i,** Ti *2p* pattern of resin surface. XPS survey spectra showed that several typical peaks for O *1s*, Ti *2p*, in the resin. The intensity of peaks at 103.18 eV correspond to the Si-O bonds while 458.63 eV and 464.48 eV correspond to the Ti-O bonds, respectively.

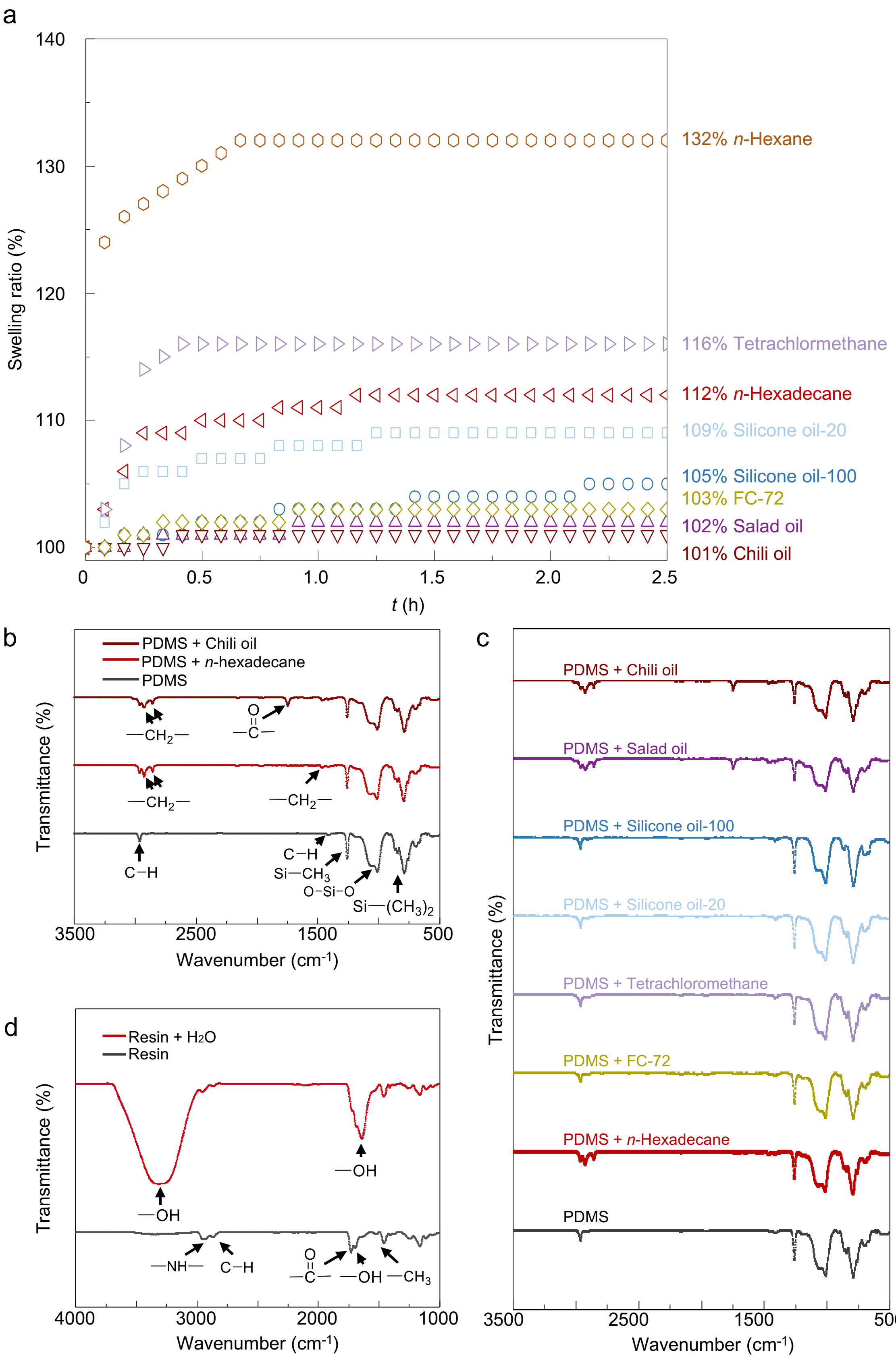

Supplementary Figure 2 | The swelling ratio of the various oil-infused PDMS surface. a, The swelling ratio of the various oil-infused PDMS surface. **b,** FTIR spectra for PDMS (black), PDMS + *n*-hexadecane (red) and PDMS + chili oil (dark red). The PDMS with the *n*-

hexadecane invasion showed two stretching vibration peaks near 2925 cm^{-1} and a bending vibration peak at 1466 cm^{-1} attributed to $-CH_2$. The PDMS with chili oil invasion showed a stretching vibration peak at 1747 cm^{-1} assigned to C=O besides the two stretching vibration peaks near 2925 cm^{-1}. **c,** FTIR spectra for the PDMS immersed with different oil phases. The FTIR spectra showed that the resin was successfully wetted by water and wetted resin showed a strong stretching vibration peak at 3305 cm^{-1} and a bending vibration peak at 1639 cm^{-1} attributed to $-OH$. **d,** FTIR spectra for resin (black) and resin + H_2O (red).

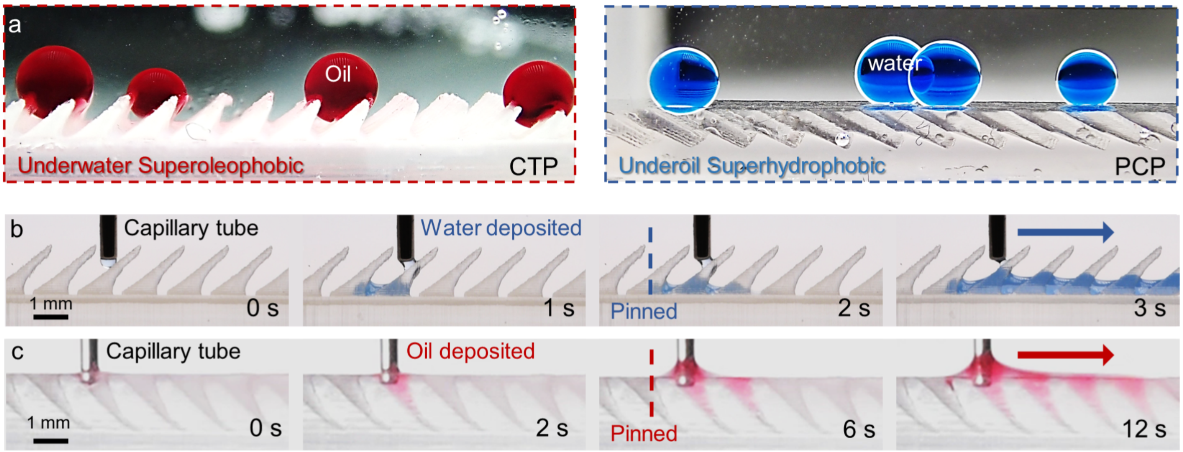

Supplementary Figure 3 | Directional transport of CTP and PCP. a, The wetting of CTP by oil under a water enviroment and PCP by water in an oil system. **b,** Directional transport of water on CTP. **c,** Directional transport of oil on PCP.

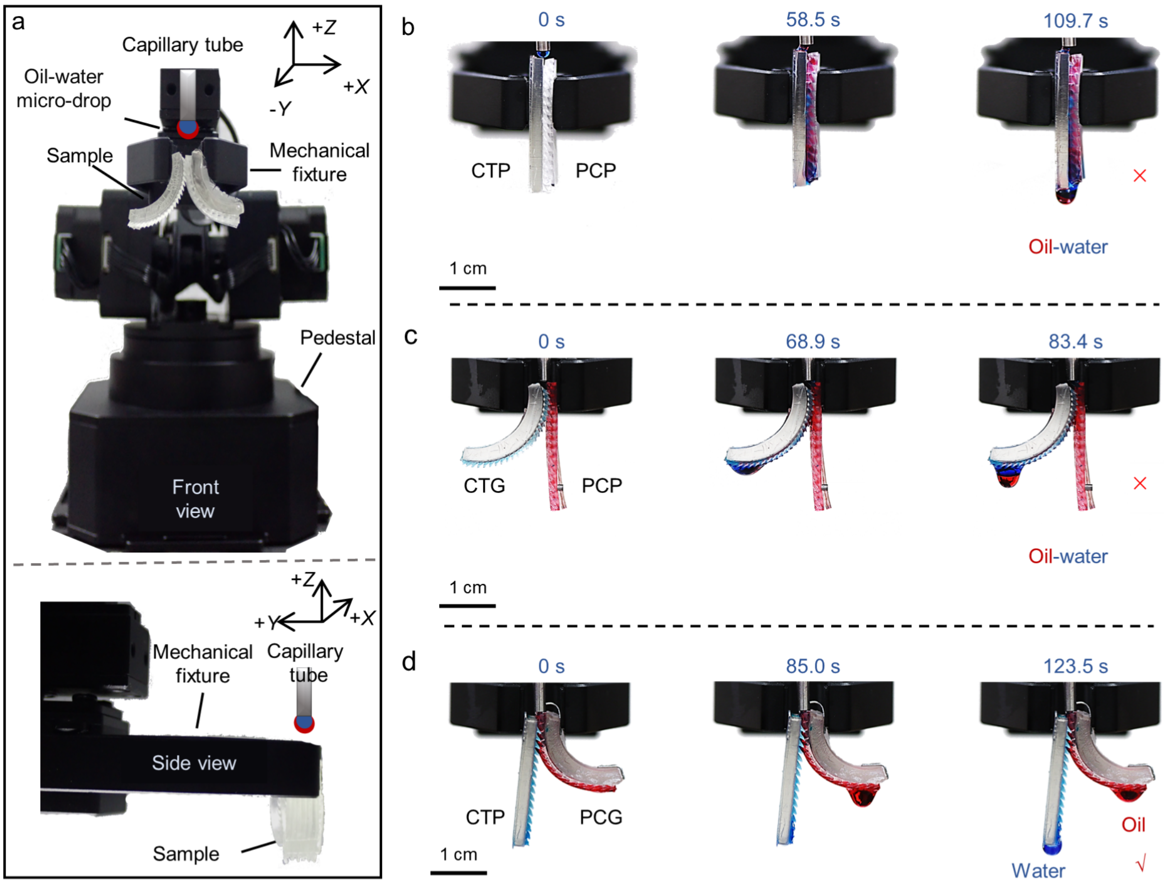

Supplementary Figure 4 | Separation of oil-water between plane and gear. a, Front and side view images of the oil-water micro-drop separation device. The samples are caught by a mechanical fixture, which can be moved in three-dimensional space by a mechanical arm controllably. **b-d,** Oil-water micro-drop separation experiments *via* wetted samples. The deposited speed (v) is 1 μL/s for blue-dyed water or red-dyed oil (n-hexadecane). So, the total deposited speed is 2 μL/s. **b,** Unwetted CTP and PCP cannot achieve oil-water micro-drop separation. Water and oil will be merged into oil-water micro-drop again at the bottom of the samples. **c,** Wetted CTG and PCP cannot achieve oil-water micro-drop separation. Both water and oil will flow along the wetted CTG. **d,** Wetted CTP and PCG can achieve oil-water micro-drop separation.

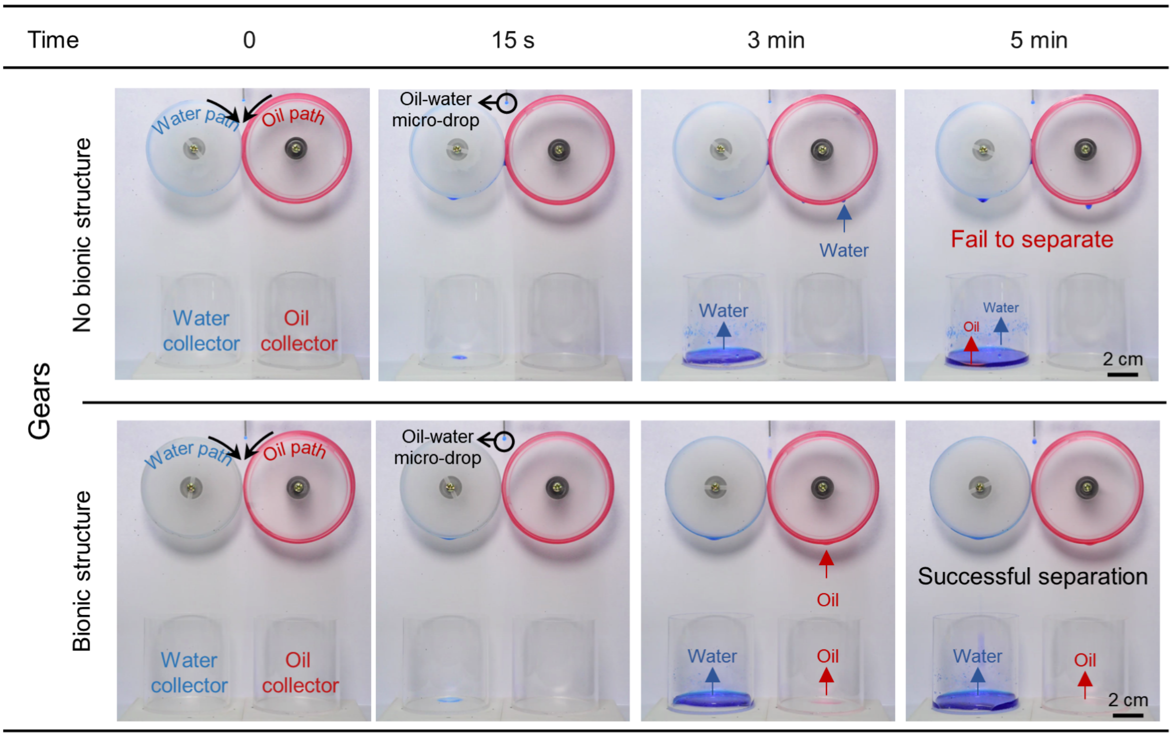

57 **Supplementary Figure 5** | Time sequence images of two gears without topology bionic features for the oil–water micro-drop separation process.

58

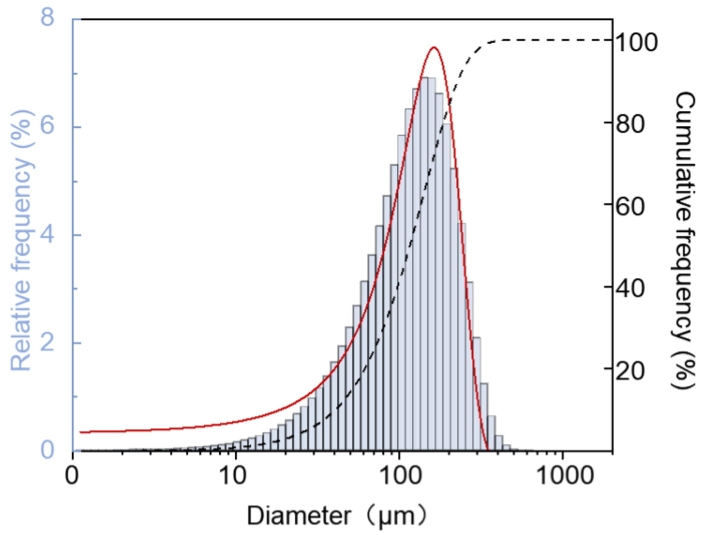

Supplementary Figure 6 | A histogram picture of emulsion particle size distribution.

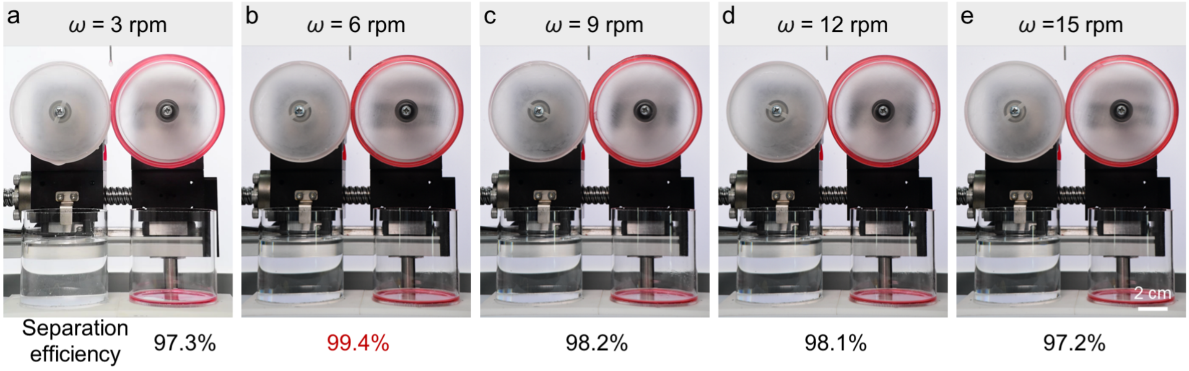

Supplementary Figure 7 | Optical sequence images of two gears for the oil–water emulsion separation process at different rotation speeds.

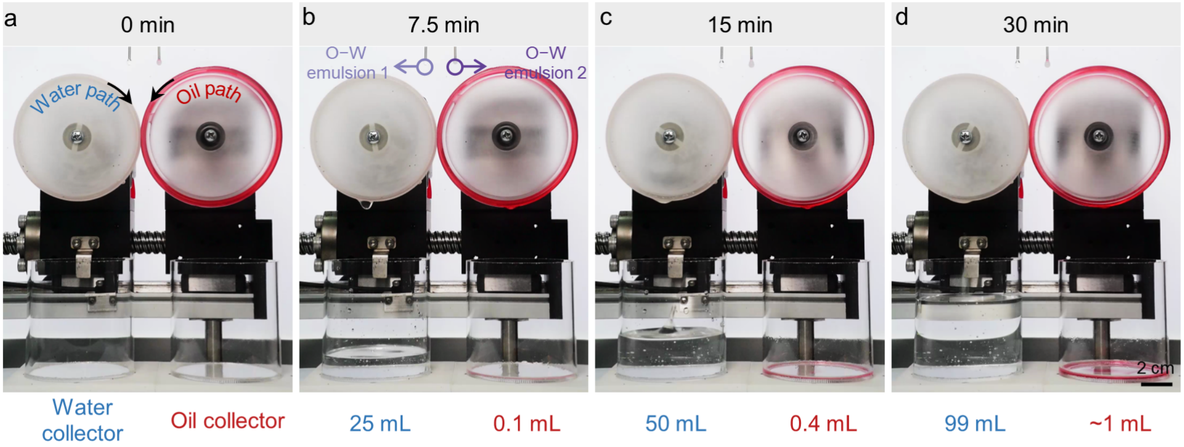

Supplementary Figure 8 | Optical sequence images of two gears for the oil–water emulsion separation with co-existing of both heavy and light oils, *i.e.,* O-W emulsion 1 system including tetrachloromethane (heavy oil, density of 1.59 g cm^{-3}) in the water phase and O-W emulsion 2 system including hexadecane (light oil, density of 0.77 g cm^{-3}) in the water phase. Both oils are dyed by red to enhance visualization.

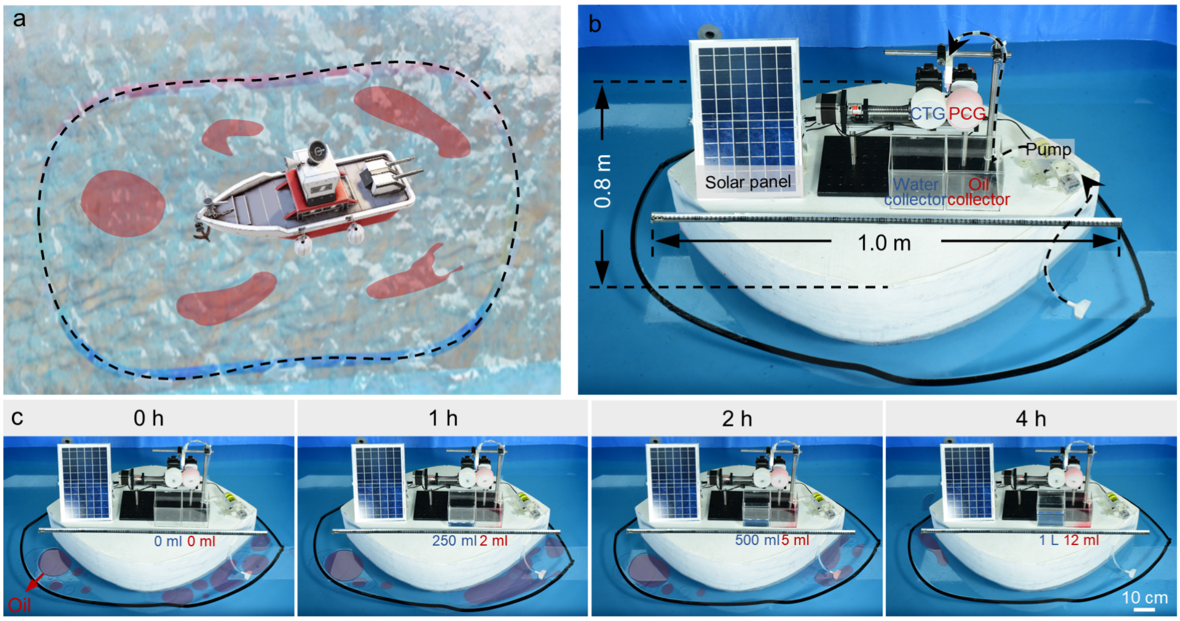

Supplementary Figure 9 | Dual-bionic superwetting gears device separating water and oil in a swimming pool. a, Schematic of the oil leaked in open waters. **b,** Optical images of the boat-integrated dual-bionic gears separator. **c,** Time sequences of the separation process for gears device to separate water and oil.

Supplementary Table 1 | Wettability of various substrates in different environments

Substrates	In air		Under oil	Under water
	WCA	OCA	WCA	OCA
Plane resin	90.3 ± 2.0°	~ 0°	142.8 ± 1.2°	63.1 ± 1.4°
CTP resin	121.1 ± 1.8°	~ 0°	138.0 ± 4.5°	91.2 ± 1.0°
Superhydrophilic plane resin	5.3 ± 0.3°	2.7 ± 0.5°	29.6 ± 8.2°	155.8 ± 1.7°
Superhydrophilic CTP resin	~ 0°	~ 0°	38.8 ± 6.5°	162.3 ± 2.2°
PDMS Plane	109.5 ± 1.2°	21.2 ± 0.4°	166.0 ± 1.1°	30.4 ± 2.0°
PCP (Oil infused PDMS)	60 ~ 80°	~ 0°	161.7 ± 0.4°	~ 0°

Supplementary Table 2 | Comparison of oil-water separation ability reported in the recent literatures with our work

Main materials	Wettability	Emulsion separation	Emulsion type	External field	Separation rate	Anti-fouling	Ref.
Hydrogel grafted PVDF	Sueprhydrophilic/ Underwater superoleophobic	√	O/W	√	25000 L m^{-2} h^{-1} bar^{-1}	√	41
TiO_2, TEOS	Superhydrophilic/ Superoleophobic	√	O/W	-	1200 L m^{-2} h^{-1}	-	54
Superamphiphilic, SiO_2-TiO_2	Superamphiphilic/ Underwater superoleophobic	√	O/W O/O	-	-	-	43
Cu, PDMS	Underwater superamphiphilic	√	O/W	-	1.128 mL cm^{-2} s^{-1}	-	19
Poly (N-isopropylacrylamide)	Hydrophilicity & underwater Superoleophobicity/ Hydrophobicity & superoleophilicity	√	O/W W/O	-	-	-	16
Fluoropolymer/SiO_2, stainless steel mesh	superhydrophobic/ Superoleophilic	√	W/O	-	-	-	42
Aluminum phosphate, TiO_2	Superamphiphilic/ Underwater superoleophobic	√	O/W	-	200 L m^{-2} h^{-1}	√	35
Poly (melamine formaldehyde), SiO_2	Sueprhydrophilic/ Underwater superoleophobic	√	O/W	-	2.5×10^5 L m^{-2} h^{-1} bar^{-1}	√	45
GO, C_3N_4, TiO_2	Sueprhydrophilic/ Underwater Superoleophobic	√	O/W	√	4536 L m^{-2} h^{-1} bar^{-1}	√	55
Acrylic acid and styrene/ divinyl benzene	Hydrophilic/ Oleophilic	√	O/W	√	-	-	47
Hydrolyzed polyacrylonitrile	Sueprhydrophilic/ Underwater superoleophobic	√	O/W	-	5152 L m^{-2} h^{-1}	√	46
Melamine sponge	Superhydrophobic/ Superoleophilic	-	W/O	-	156 700 L m^{-2} h^{-1} bar^{-1}	-	44
Divinylbenzene, PVDF	Superhydrophobic/ Superoleophilic	-	W/O	√	1500 L m^{-2} h^{-1}	-	56
Attapulgite, PVDF	Sueprhydrophilic/ Underwater Superoleophobic	-	O/W	-	360 L m^{-2} h^{-1} bar^{-1}	-	36
Photocurable resin PDMS	Superhydrophobic/ Superoleophilic/ Under-water superoleophobic/ Under-oil superhydrophobic	√	O/W	-	> 2000 L m^{-2} h^{-1}	√	This work

Legend for movies

Supplementary Movie 1. The designed dual-bionic model with millimeter-scaled and micro-scaled structures steers liquid spreading and separation.

Supplementary Movie 2. One-hour oil-water separation process.

Supplementary References

1. Li, F., Wang, Z., Huang, S., Pan, Y., & Zhao, X. Flexible, durable, and unconditioned superoleophobic/superhydrophilic surfaces for controllable transport and oil–water separation. *Adv. Funct. Mater.*, **28**, 1706867 (2018).
2. Liu, Y., et al. 2D heterostructure membranes with sunlight-driven self-cleaning ability for highly efficient oil–water separation. *Adv. Funct. Mater.*, **28**, 1706545 (2018).
3. Zhang, W., et al. A solvothermal route decorated on different substrates: Controllable separation of an oil/water mixture to a stabilized nanoscale emulsion. *Adv. Mater.* **27**, 7349-7355 (2015).
