# Supplementary material for: Dual-bionic superwetting gears with liquid directional steering for oil-water separation
Source: Nat Commun. 2023 Jul 12;14:4128. doi: 10.1038/s41467-023-39851-1 (PMC10338494; doi:10.1038/s41467-023-39851-1)
Supplement: Supplementary file 3 — Description of Additional Supplementary Files [file 41467_2023_39851_MOESM3_ESM.pdf]

Description of Additional Supplementary Files

File Name: Supplementary Movie 1
Description: The designed dual-bionic model with millimeter-scaled and micro-scaled structures steers liquid spreading and separation.

File Name: Supplementary Movie 2
Description: One-hour oil-water separation process.
